# Neurogenic and non-neurogenic functions of endogenous neural stem cells

**DOI:** 10.3389/fnins.2014.00092

**Published:** 2014-04-29

**Authors:** Erica Butti, Melania Cusimano, Marco Bacigaluppi, Gianvito Martino

**Affiliations:** Neuroimmunology Unit, Division of Neuroscience, Institute of Experimental Neurology, San Raffaele Scientific InstituteMilan, Italy

**Keywords:** neural stem cells, neurogenesis, inflammation, transplantation, germinal niches, bystander effect

## Abstract

Adult neurogenesis is a lifelong process that occurs in two main neurogenic niches of the brain, namely in the subventricular zone (SVZ) of the lateral ventricles and in the subgranular zone (SGZ) of the dentate gyrus (DG) in the hippocampus. In the 1960s, studies on adult neurogenesis have been hampered by the lack of established phenotypic markers. The precise tracing of neural stem/progenitor cells (NPCs) was therefore, not properly feasible. After the (partial) identification of those markers, it was the lack of specific tools that hindered a proper experimental elimination and tracing of those cells to demonstrate their terminal fate and commitment. Nowadays, irradiation, cytotoxic drugs as well as genetic tracing/ablation procedures have moved the field forward and increased our understanding of neurogenesis processes in both physiological and pathological conditions. Newly formed NPC progeny from the SVZ can replace granule cells in the olfactory bulbs of rodents, thus contributing to orchestrate sophisticated odor behavior. SGZ-derived new granule cells, instead, integrate within the DG where they play an essential role in memory functions. Furthermore, converging evidence claim that endogenous NPCs not only exert neurogenic functions, but might also have non-neurogenic homeostatic functions by the release of different types of neuroprotective molecules. Remarkably, these non-neurogenic homeostatic functions seem to be necessary, both in healthy and diseased conditions, for example for preventing or limiting tissue damage. In this review, we will discuss the neurogenic and the non-neurogenic functions of adult NPCs both in physiological and pathological conditions.

## Introduction

In 1913 Santiago Ramón y Cajal established that neurons of the brain are only generated during the neurodevelopmental phase, thus setting the so called “no new neurons” doctrine (Ramon Y Cajal, 1913). However, he soon reconsidered his conclusions when evaluating the results of an experiment performed a couple of years before by his younger assistant Francisco Tello. This experiment, in fact, showed that regenerating fibers growing from the stump of a transected optic nerve could suture with a “regenerating” peripheral sciatic nerve (Tello, 1907).

Nevertheless, despite this initial hint, the existence of dividing cells of neural origin in the central nervous system (CNS) was still debated (Hamilton, 1901; Allen, 1912) and could not be formally demonstrated until the beginning of the'60 when Smart (1961) and Altman (Altman and Das, 1965) demonstrated the effective presence of proliferating neural cells—i.e., neurogenesis—in the adult rodent brain. However, this finding would have been indisputably confirmed only 20 years later, namely when Fernando Nottebohm showed that neurogenesis in the ventricular zone is a phenomenon that normally occurs in intact adult female canaries (Nottebohm, 1981; Goldman and Nottebohm, 1983). Few years' later, adult neural stem/precursor cells (NPCs) were identified as a source of new neurons also in the brain of non-human primates and humans (Kukekov et al., 1999; Ming and Song, 2005). Later on, *in vitro* stable culturing systems either for rodent and human NPCs were established (Reynolds and Weiss, 1992).

Nowadays, we know that neurogenesis in the adult brain occurs in physiological conditions in specific neurogenic niches that have particular anatomical and functional characteristics. The role of neurogenesis after injury however still needs to be fully clarified. While there is substantial evidence that active, and latent, neurogenic niches might contribute to the formation of new cells upon CNS tissue damage, the precise role of these newly formed cells has not been yet completely understood. Here we review the possibility that endogenous NPCs exert functional roles not directly related to the production of new cells (the so called “non-neurogenic functions”).

## Neurogenesis in the adult brain: from cells to functions

### The adult rodent brain

Neurogenesis in the adult rodent brain occurs during adulthood in two main neurogenic niches, namely in the subgranular zone (SGZ) of the dentate gyrus (DG) in the hippocampus and in the subventricular zone (SVZ) of the lateral ventricles.

The SGZ is a thin layer of cells located between the two DG layers of granule and hilus cells. The primary role of SGZ is to generate new cells capable to functionally integrate within the DG granule layer. The DG granule layer is mainly composed by primary excitatory neurons supporting cognitive functions, such as memory and learning (Shors et al., 2002; Zhao et al., 2008). The development of granule cells from NPCs proceeds throughout different intermediate steps (Filippov et al., 2003). NPCs first develop into (i) radial astrocytes (i.e., type I cells) that, in turn, generate (ii) intermediate neural progenitors (i.e., type-D cells or type II progenitors) (Fukuda et al., 2003)—which are immature progenitors (also called neuroblasts) further differentiating into (iii) neuroblasts. Neuroblasts can be further sub divided into D1 (immature) and D2 (more differentiated) cells (i.e., type G or type III cells) (Filippov et al., 2003; Zhao et al., 2008), which progressively acquire electrophysiological characteristics of granule neurons. SGZ neurogenesis occurs in parallel to angiogenesis (Palmer et al., 2000) and endothelial cells act as scaffolding cells for NPCs. Therefore, endothelial cells provide signals and soluble factors that favor angiogenesis but also neurogenesis (Riquelme et al., 2008).

The second main neurogenic niche is the SVZ, a region located in the lateral side of the two lateral ventricles. This region originates from the neuroventricular epithelium of the embryonic ventricular zone, the area where radial glia proliferates during development. Similarly to the SGZ, the SVZ shows a rather heterogeneous population of stem and progenitor cells. Here we can find (i) relatively quiescent stem cells, known as B cells, that give rise to (ii) actively proliferating cells representing intermediate progenitors in transit to the terminal differentiation (i.e., type C cells or transit amplifying cells) (Doetsch et al., 1999). Type C cells differentiate into (iii) neuroblasts (i.e., immature type A cells) that migrate along the rostral migratory stream (RMS) toward the olfactory bulb (OB) to give rise to new OB granule cells (Lois and Alvarez-Buylla, 1994; Belluzzi et al., 2003). The SVZ can be subdivided anatomically into three main structural domains: domain I (wall of the ventricle) contains ependymal cells as well as the primary cilium of type B cells and is in direct contact with the cerebrospinal fluid (CSF); domain II (below the wall of the ventricle) contains the cell bodies of type-B cells, type C cells, type A cells, neuronal terminals, and other supporting cells; domain III contains basal processes of B-cells that terminate in specialized end-feet capable of contacting blood vessels (Fuentealba et al., 2012). Due to their anatomical localization SVZ NPCs are strategically positioned within the brain: on the one hand, they are in direct contact with the CSF through their apical processes, and, on the other hand, they are tightly apposed to blood vessels forming a peculiar “periventricular” blood brain barrier (BBB) that is the barrier circumventing the lateral ventricles and the third and the fourth ventricle. SVZ NPCs are thus in close communication with two different peripheral blood-related microenvironments (Sawamoto et al., 2006; Mirzadeh et al., 2008; Tavazoie et al., 2008). It is still matter of debate whether the periventricular BBB is more permeable thus facilitating type B and C cells to receive blood-borne molecules regulating self-renewal and differentiation. Apart from the blood compartment, the SVZ is also located very close to crucial areas of the forebrain (i.e., basal ganglia, striatum) that contain GABAergic neurons capable of modulating interconnections between several cortical and sub-cortical brain areas (Koos and Tepper, 1999). In fact, NPCs in the SVZ are separated from the caudate nucleus and the striatum only by a layer of myelin and are in intimate contact with surrounding glia and blood vessels (Doetsch et al., 1999; Alvarez-Buylla and Lim, 2004). This peculiar position makes SVZ NPCs susceptible to the action of several neurotransmitters such as GABA (Platel et al., 2008, 2010), glutamate (Platel et al., 2010), ATP (Abbracchio et al., 2009), and acetylcholine (Cooper-Kuhn et al., 2004; Young et al., 2011), all neurotransmitters released from nearby neurons and collaterals. It is highly likely that SVZ NPCs can be directly influenced by the activity of neuronal networks (Tong et al., 2014). The decreased proliferation of NPCs, so far observed in Parkinson's disease, has been attributed to the loss of dopaminergic innervation of the SVZ (Curtis et al., 2007a). *Post-mortem* studies in humans have identified dopaminergic fibers in contact with epidermal growth factor receptor (EGFR)- positive cells in the SVZ (Hoglinger et al., 2004). In addition, the SVZ area is innervated by serotoninergic fibers (Diaz et al., 2009) and serotonin has been documented to increase neurogenesis in the SVZ (Encinas et al., 2006; Kazanis, 2009).

### The adult human brain

Although it has been variably shown that the two main neurogenic regions of the rodent brain, the SGZ and the SVZ, are also present in the adult human brain, human neurogenesis has some peculiarities that need to be highlighted.

In the 1990s, a study by Eriksson and colleagues performed in a group of cancer patients receiving the DNA labeling nucleotide Bromodeoxyuridine (BrdU) showed the BrdU signal in hippocampal neurons (Eriksson et al., 1998). This work formally established the presence of adult neurogenesis in the human hippocampus during adulthood. However, the observed neurogenesis could also have been attributed to the underlying pathology. Some years later Knoth et al. (2010) confirmed the presence of neurogenesis in the adult human hippocampus based on data obtained from 54 human autoptic specimens (age 0–100). In the same study, qualitative and quantitative age-related changes—very similar to those occurring in the rodent hippocampus—further confirmed and expanded these findings (Knoth et al., 2010).

The human SVZ behaves, instead, very differently to the human adult hippocampus. In this region, the extent of this continuous neurogenesis as well as the presence of a RMS is still matter of debate. In 2004, Sanai and co-workers described within the SVZ a ribbon of proliferating astrocytes—lining the lateral ventricles of the adult human brain—that behaved as multipotent progenitor cells *in vitro* (Sanai et al., 2004). However, they did not find any evidence of chains of migrating neuroblasts in the SVZ or in close proximity to the OB (Sanai et al., 2007). After this provocative works, an intense debate occurred about the existence of a human RMS. In 2007, Curtis and colleagues showed histological evidence of a human RMS-like structure organized around a lateral ventricular extension reaching the OB (Curtis et al., 2007a,b; Sanai et al., 2007). Two successive reports challenged the existence of a RMS; only a ventromedial prefrontal cortex stream was observed in infants up to 2 years of age but not in adults (Sanai et al., 2011). More recently, a retrospective ^14^C birth dating study showed that there is rather minimal adult neurogenesis in the human OB (Bergmann et al., 2012). While in the adult human brain 700 new neurons are added in each hippocampus per day (corresponding to an annual turnover of 1.75% of the neurons within the renewing fraction), OB neurons are as old as the individual since the decrease in neuroblast numbers in the SVZ and their migratory path suggested that there is negligible, if any, adult OB neurogenesis in humans (Spalding et al., 2013). It was concluded that less than 1% of the OB adult human neurons are exchanged over a century.

Therefore, while in the human brain the presence of neurogenic niches in the hippocampus and in the SVZ seems to be similar to other mammals, the precise function, role and fate of periventricular generated neuroblasts needs to be further refined.

### Studying the role of endogenous NPCs in the adult brain

While the study of neurogenesis was initially hampered by the technical limitation of tracing and labeling the dividing cells (Ming and Song, 2005), in the following years the major challenge became the inability to specifically ablate NPCs in a determined neurogenic niche. Consequently, approaches to eliminate proliferating cells, using either intracerebral administration of antimitotic drugs, such as Arabinofuranosyl Cytidine (AraC), or brain x-rays irradiation, were developed (Doetsch et al., 1999; Monje et al., 2003) (Table 1). 60–90% of proliferating cells within neurogenic niches can be successfully ablated by using the x-rays irradiation method (Kageyama et al., 2012). However, there are still limitations due to the overt inflammatory reactions caused by the procedure (Palmer et al., 2000). These methods are however not selective for NPCs as also other proliferating cells (e.g., microglia, pericytes) might be affected by the treatment.

**Table 1:** **Newly developed mouse models to study the role of neurogenesis by specific ablation of different types of NPCs**.

**Mouse model**	**Target niche and cells**	**Treatment**	**Ablation efficacy**	**Experimental model**	**References**
Nestin-δ-HSV-TK-EGFP	SVZ and SGZ	4 weeks of GCV	≈30% in SVZ	Distal MCAO	Sun et al., 2013
			≈60% in SGV		
FoxJ1-CreER; floxed K-Ras	Ependymal cells	5 days of tamoxifen	≈90%	Spinal cord injury	Sabelström et al., 2013
Nestin-TK	SVZ	4 weeks of GCV	≈70%	Stroke and epilepsy	Butti et al., 2012
Dcx-TK	SVZ and SGZ	2 weeks of GCV	≈80%	Permanent MCAO	Jin et al., 2010; Wang et al., 2012
GFAP-TK	SGZ	4–12 weeks of GCV	≈99%	Moderate stress diseases	Snyder et al., 2011
Nestin-TK	SVZ and SGZ	4 weeks of GCV	≈90%	Study of neurogenesis	Singer et al., 2009
Wt rats and mice	SGZ	X-ray irradiation	≈85%	Study of hippocampal function	Kitamura et al., 2009
Nes-CreERT2; NSE-DTA	SVZ and SGZ	4 days of tamoxifen	≈30%	Study of olfactory bulbs	Imayoshi et al., 2008

*Dcx, doublecortin; EGFP, enhanced green fluorescent protein; GCV, ganciclovir; GFAP, green fibrillary acidic protein; HSV, herpes simplex virus; MCAO, middle cerebral artery occlusion; NSE-DTA, neuron-specific enolase 2–diphtheria toxin fragment A; SVZ, subventricular zone; SGZ, subgranular zone; TK, thymidine kinase*.

Recently, transgenic animals have been developed using various types of “marker” genes selectively expressed by NPCs or their progeny. Among transgenic models those based on the transgenic use of the thymidine kinase (TK) gene under the promoter of the GFAP (Garcia et al., 2004), Nestin (Singer et al., 2009), or Doublecortin (Jin et al., 2010) are the most used so far along with models in which the selective expression in NPCs of the diphtheria toxin fragment A (DT-A), under the neuron-specific enolase 2 gene promoter, has been achieved (Imayoshi et al., 2008). Another method to stop the proliferation of NPCs without direct ablation of the cells was the creation of conditional mouse models relying on the cre/flox system such ad the FoxJ1-CreER:floxed K-Ras mouse. In this model, FoxJ1 positive cells (i.e., NPCs and ependymal cells in the CNS), upon administration of tamoxifen, are deleted for the Ras genes that are required for mitosis (Sabelström et al., 2013). Transgenic models are efficacious since 80–90% NPC ablation can be obtained; however, also in these models it is not possible to selectively analyse one or the other neurogenic niche. This can be resolved by using a model that has been recently published by our group and consists in a selective ablation of SVZ NPCs upon GCV administration due to the presence of the transgene only in the SVZ (Butti et al., 2012).

Finally, neurogenesis can be also studied in aged mice. In fact, in those mice a physiological decline of neurogenesis occurs both in the hippocampus and in the SVZ. Ageing does affect all tissues, but at the same time the decline of neurogenesis in this model is a naturally occurring process and therefore does not require a specific gene manipulation (Villeda et al., 2011).

## Role of endogenous NPCs in physiological conditions

### Neurogenic functions

After having been a debated issue for years, it is now clear that hippocampal neurogenesis is a necessary process to preserve spatial memory, to support memory acquisition, especially in the early period of memory formation, (i.e., recent memory), and in the maintenance of the overall memory capacities (Snyder et al., 2005; Imayoshi et al., 2008). Sahay and colleagues showed that increased hippocampal neurogenesis—obtained in mice with the apoptosis-inducing gene Bax conditionally ablated from NPCs (i.e., Baxfl/fl Nes-CreERT2 mice) —was paralleled by an increased behavioral performance during a specific cognitive task where two similar contexts needed to be distinguished (Sahay et al., 2011). Kitamura and colleagues showed that hippocampal neurogenesis, particularly concerning the integration of new neurons, is a key factor in the gradual decay of DG long term potentiation (LTP) (Kitamura et al., 2009). They also showed that decreased neurogenesis is accompanied by a prolonged hippocampus-dependent period of associative fear memory: this mechanism has been proposed to play a role in clearing disused old memories to preserve the learning capacity of the hippocampus (Willshaw and Buckingham, 1990). Animals exposed to an environmental enrichment showed enhanced hippocampal neurogenesis (Kempermann et al., 1997).

The functional role of NPCs residing within the SVZ is certainly more controversial. As said before, newly formed NPCs in the rodent SVZ migrate along the RMS to the OB where they integrate as interneurons within the granule and glomerular cell layers; a process considered important in maintaining and reorganizing the OB system (Imayoshi et al., 2008). The integration of the new neurons in the OB and DG is varied: in the OB, neurogenesis contributes to the maintenance and reorganization of the whole system while in the DG new neurons are added to modulate and refine the existing neuronal circuits (Imayoshi et al., 2008). While SVZ neurogenesis in the adult brain seems not to exert a role in retaining the memory of spontaneous odor discrimination and innate olfactory preference (Imayoshi et al., 2008), it seems to be involved in consolidating long-lasting olfactory traces (Gheusi et al., 2000; Lazarini et al., 2009). Indeed, the increased survival of new-born granule cells observed after the enrichment is necessary for the increased inhibitory activity in the OB and leads to a better discrimination of highly similar odorants (Moreno et al., 2009). Recent studies have confirmed these data and assessed that while easy odor tasks (Mandairon et al., 2006)—e.g., the habituation- dishabituation test—do not need neurogenesis (Kageyama et al., 2012), more difficult odor tasks, instead, do require modulation of the new-born neuron survival (Mandairon et al., 2006).

Besides the role in smell recognition, the instinctive response to pheromones is also processed by the main and accessory olfactory systems; SVZ neurogenesis plays an essential role in this context (Kageyama et al., 2012). For example, olfactory activities are very important for the maintenance of pregnancy (Bruce, 1959; Kaba et al., 1994): pregnancy induces biphasic stimulation of neurogenesis in the SVZ, leading to a biphasic increase in the production of both granule cells and periglomerular cells in the OB (Shingo et al., 2003). Neurogenesis in females is also induced by dominant male pheromones and seems to be important for sexual behaviors (Mak et al., 2007). Also in male, paternal-offspring recognition behaviors seem to rely on postnatal offspring interaction and are coupled to increased neurogenesis in the paternal OB and hippocampus (Mak and Weiss, 2010; Kageyama et al., 2012). Finally, SVZ neurogenesis might be required for predator avoidance and sex-specific responses that are olfaction dependent and innately programmed (Sakamoto et al., 2011).

### Non-neurogenic functions

In the last few years, other non-neurogenic functions of NPCs in the brain have been unraveled. NPCs are in fact able to produce and secrete a wide variety of factors that regulate and drive complex functions of the brain. A recent report showed that neuroblasts derived from both neurogenic niches (the SVZ and SGZ) exert a physiological phagocytic activity in clearing apoptotic neuronal precursors, and that this phagocytic activity is critically important in maintaining neurogenesis in the brain. Interestingly, NPC phagocytosis requires the intracellular engulfment protein ELMO1 to promote Rac activation downstream of phagocytic receptors (Lu et al., 2011).

Moreover, recent evidence supports the importance of non-neurogenic functions of NPCs. Sierra et al. demonstrated in fact that apoptotic new-born cells are rapidly cleared out through phagocytosis by unchallenged microglia present in the adult SGZ niche and that microglia is important in maintaining the homeostasis of the baseline neurogenic cascade (Sierra et al., 2010). Mosher et al. expanded this finding by demonstrating that NPCs are able, through the secretion of vascular endothelial growth factor (VEGF), to modulate microglial activation, proliferation and phagocytosis (Mosher et al., 2012). Furthermore, a bilateral cross-talk between NPCs and microglia seems to take place (Mosher et al., 2012).

Another “homeostatic” function coupled to NPCs has been recently described. Despite not having classical features of a neurogenic niche, median eminence tanycytes may also generate new-born neurons (Kokoeva et al., 2005; Lee et al., 2012). After a first study supporting the idea that hypothalamic neurogenesis in adult mice has a role in the control of energy-balance, including the capacity of regulating leptin-induced phosphorylation of signal transducer and activator of transcription 3 (STAT3) (Kokoeva et al., 2005), another recent work showed that median eminence tanycytes have a role in regulating the weight and metabolic activity of adult mice (Lee et al., 2012).

Moreover, newly generated neuroblasts residing within the SGZ seem to be able to dynamically regulate stress reactivity at both the endocrine and behavioral levels by buffering stress responses, through the regulation of the hypothalamic–pituitary–adrenal axis (Snyder et al., 2011). In fact, neurogenesis-deficient mice also showed increased food avoidance after acute stress, increased behavioral despair in the forced swim test, and decreased sucrose preference, a measure of anhedonia (Snyder et al., 2011). It would be interesting to understand whether the observed alterations can be attributed to an alteration of median eminence tancytes (and vice versa), given that the models used for ablation of NPCs in this work did not exclusively target a single NPC subpopulation.

**Figure 1:**
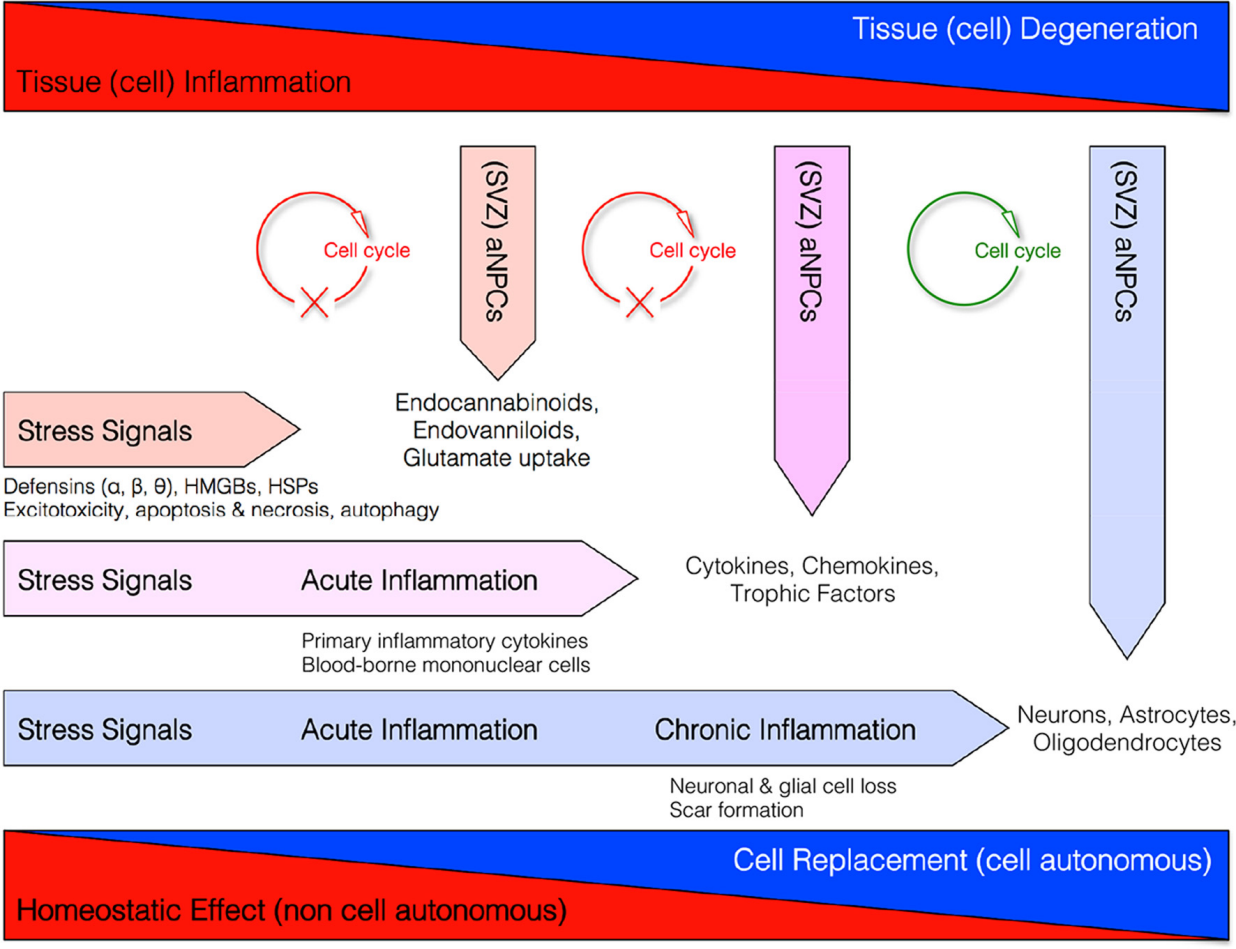
**Homeostatic multi-step actions exerted by endogenous NPCs: from maladaptive (stressful) conditions to pathological chronic tissue damage**. Endogenous NPCs adapt their homeostatic functions to the needs of the tissue. In order to reduce excitotoxicity so to prevent reactive inflammation, endogenous NPCs release neuroprotective molecules (i.e., endocannabinoids, endovanilloids) and increase glutamate uptake as soon as the occurrence of early stress signals. If this barrier fails and acute inflammation occurs, endogenous NPCs release different neuroprotective and anti-inflammatory molecules (e.g., cytokines, chemokines, and trophic factors) that, in turn, restrain the CNS infiltration of blood-borne inflammatory cells and the acute inflammatory reaction. This latter second-step process is also finalized to reduce the secondary tissue damage. Finally, during chronic inflammatory conditions when tissue architecture is already compromised, NPCs might differentiate into new cellular elements in order to replace endogenous cells lost.

These data altogether support the concept that NPCs might exert, besides pure neurogenic functions, also a broad spectrum of “bystander” non-neurogenic functions aimed at maintaining the homeostasis of the brain (Figure 1) (Martino and Pluchino, 2006).

## Role of endogenous NPCs during CNS pathology

### Neurogenic functions

Different types of brain damage—such as stroke, epileptic seizures, trauma—induce the proliferation of NPCs in neurogenic areas, i.e., the SGZ and the SVZ (Riquelme et al., 2008). The majority of neurons formed in SGZ after an insult become dentate granule cells, similar to what occurs in the intact brain, while in the SVZ newly generated cells often migrate, away from the RMS, toward the lesion site (Jin et al., 2001).

Adult brain reacts to an ischemic injury by a long-lasting generation of neuroblasts from the SVZ; SDF-1a/CXCR4 signaling regulates the migration of new striatal neurons generated from endogenous NPCs toward the ischemic damage (Imitola et al., 2004). Within these newly formed peri-infarct neurovascular niches, newly-born immature neurons interact with the remodeling vasculature thanks to their production of stromal-derived factor 1 (SDF1) and angiopoietin 1 (Ang1) (Ohab et al., 2006). Interestingly neurogenesis and angiogenesis, another important reparative process taking place in the peri-ischemic tissue, are tightly coupled after stroke by VEGF that stimulates cell genesis (Teng et al., 2008).

A long-lasting neurogenic response reactive to ischemic injury has been observed not only in animal models but also in stroke patients (Marti-Fabregas et al., 2010). Interestingly, the enhanced neurogenic response is paralleled by increased microglia recruitment, probably due to a stroke-induced up regulation of CXCL10 in the SVZ; this chemokine might act as chemoattractant of CXCR3-expressing microglia (Rappert et al., 2004; Thored et al., 2009). About 80–90% of newly formed striatal neurons that potentially could replace the dead neurons will eventually die (Arvidsson et al., 2002; Thored et al., 2006, 2007). In fact, only a small portion of SVZ-derived cells migrated into striatum does assume features of mature neurons with action potentials in a rodent model of ischemic stroke (Arvidsson et al., 2002).

Similarly to stroke, also epilepsy is associated with an increased level of progenitor proliferation paralleled with an accelerated maturation and integration of only few newly generated neurons (Rotheneichner et al., 2013). Neurons formed in the DG, after an epileptic insult, undergo caspase-mediated apoptotic death, similarly to NPCs isolated from the adult SVZ (Ekdahl et al., 2001). In animal models of epilepsy, the initial rise in neurogenesis is then followed by a long-lasting reduction of neurogenesis (Hattiangady et al., 2004). Reduced cell proliferation has been also observed in the hippocampus of children during the chronic phase of a frequent seizure convulsive disorder (Mathern et al., 2002; Rotheneichner et al., 2013). In experimental epilepsy, SVZ-derived cells migrate toward the hippocampus and differentiate terminally into glial but not neuronal cells (Parent et al., 2006). Newly born neurons might exacerbate chronically epileptic hippocampus if they aberrantly migrate and incorporate in the dentate hilus (Hattiangady and Shetty, 2008). Interestingly, inflammation might influence the functional integration of adult-born hippocampal neurons as a high degree of synaptic plasticity of the new neurons has been reported in an inflammatory environment. This effect seems to be finalized to counteract inflammation-induced increase of excitatory input (Jakubs et al., 2008). However, the extent to which seizure-induced neurogenesis might contribute to the formation of newly formed neurons destined to integrate into the damaged epileptic hippocampus still need to be clarified.

### Non-neurogenic functions

As said above, there is an increased reactive neurogenesis followed by a scarce integration of newly formed neurons into neuronal damaged circuits. This chain of events appears to be paradoxical. Several hypotheses have been proposed. One of these states that NPCs might exert tissue protective functions by deviating from their neuronal default into a glial differentiation pathway or remaining undifferentiated and secrete neuroprotective molecules in a bystander fashion.

Instead of differentiating into the neuronal default pattern, NPCs (from both SVZ and SGZ) may turn into both astroglial and oligodendroglial cells—a gliogenic rather than a neurogenic response—in order to constrain and/or prevent tissue damage. Several recent evidence supports this NPC-mediated phenomenon reacting to a CNS injury.

Localized photothrombotic/ischemic cortical injury triggers the production of BBB stabilizing astrocytes from the postnatal SVZ niche; an event controlled by the Notch modulator thrombospondin 4 (Thbs4). Indeed, knockout mice for Thbs4 had a distorted neuroblast-astrocyte production, an abnormal glial scar formation, and a significant delayed increase of perilesional microvascular hemorrhages (Benner et al., 2013).

In demyelinating diseases, such as multiple sclerosis (MS), NPCs in the rodent SVZ niche become activated, upon demyelination, and provide a potential source of myelinating oligodendrocytes. SVZ-derived cells expand and migrate to the lesions, undergo oligodendrogenesis (Nait-Oumesmar et al., 1999), acquire morphology of myelinating cells, and express myelin proteins (Menn et al., 2006).

Using genetic fate mapping, it has been shown that, after a spinal cord injury (SCI), ependymal cells lining the central canal of the spinal cord have neurogenic potential. Indeed, in mice undergoing SCI, ependymal cell progeny starts migrating from the ependymal layer toward the injury site within 3 days after the injury; once within the lesion site, proliferating cells predominately differentiate into scar-forming astrocytes (Barnabe-Heider et al., 2010). In fact the glial scar that forms after SCI is composed by resident astrocytes and, in its central part, by ependymal cell–derived astrocytes (Barnabe-Heider et al., 2010). Ependymal cell–derived astrocytes might thus contribute to reinforce the injured spinal cord thus avoiding the expansion of the cystic cavity (Barnabe-Heider et al., 2010). Finally, cells recruited by the SCI not only produce scar-forming glial cells, but also, to a lesser degree, oligodendrocytes (Meletis et al., 2008).

The production of new neuronal or glial cells seems not to be the prevailing and sole mechanism of reactive neurogenesis occurring in response to tissue damage.

In stroke, not integrating newly formed SVZ-derived cells seem to protect from tissue injury through the secretion of neurotrophic factors (Jin et al., 2010; Wang et al., 2012; Sun et al., 2013). In a recent work SVZ NPCs were indeed shown to protect striatal neurons from glutamatergic excitotoxicity (as that occurring in the early phase of ischemic stroke and epilepsy) by releasing endogenous endocannabinoids (AEA and 2-AG) capable of binding to their specific receptors (CB1 and CB2) (Butti et al., 2012). Interestingly endovanilloids secreted by SVZ NPCs were found to suppress the growth of high-grade astrocytomas (HGA). NPCs by releasing endovanilloids activate the transient receptor potential vanilloid subfamily member-1 (TRPV1) on HGA cells that, in turn, triggers tumor cell death and prolongs overall survival time of the mice (Stock et al., 2012).

Also in another CNS injury model, SCI, scar stabilizing NPC-derived astrocytes do not only restrict secondary enlargement of the lesion and further axonal loss (Sabelström et al., 2013), but also exert a non-neurogenic action via the secretion of growth factors acting as neuroprotectant to enhance the survival of neurons adjacent to the traumatic lesion.

As previously pointed out, whether a homeostatic function of the endogenous NPCs might occur, the SVZ zone seems to be the more appropriate area. In fact, as stated before, SVZ NPCs are in close communication with two different microenvironments being tightly apposed to blood vessels and in contact with the CSF, and also very close to crucial areas of the midbrain containing GABAergic neurons. A further confirmation of this working hypothesis came from a recent work showing that dendritic cell (DC) traffic within the CNS—from the choroid plexus to the cervical lymph nodes—along the RMS in order to modulate CNS-infiltrating regulatory T cell (Treg) function. This migration of DC seems to dampen experimental CNS auto-inflammatory diseases, thus suggesting that it ultimately prevents pathogenic T-cells from entering the CNS (Mohammad et al., 2014).

## Neurogenic vs. non-neurogenic functions

Another important, but so far only partially solved issue, concerns how of NPCs can determine their fate between neurogenic and non-neurogenic functions in pathological conditions. The predominant view, supported by NPC transplantation studies, but confirmed to be valid also for endogenous NPCs as well, is that inflammation is in part responsible for the fate decision of newly formed NPCs.

Inflammation, as process occurring as a consequence of autoimmunity and/or traumatic and ischemic injuries, alter endogenous NPC proliferation and differentiation characteristics in a non-cell autonomous fashion (Pluchino et al., 2005). When inflammation fades away and neurodegeneration prevails, endogenous NPCs tend to differentiate into multiple neuronal lineages, depending on the situation, partially capable of integrating into damaged neuronal circuits (Kokaia et al., 2012).

However, in acute inflammatory conditions, while remaining undifferentiated, transplanted SVZ-derived NPCs might promote CNS tissue healing via the secretion of immunomodulatory and neuroprotective molecules, capable of reducing detrimental tissue responses. Instead, in chronic inflammatory conditions NPCs seem to be driven toward cell replacement (Martino and Pluchino, 2006).

### Transplantation of NPCs in inflammatory CNS diseases

As underlined before, NPC replacement-based studies allow to investigate the multimodal—neurogenic vs. non-neurogenic functions—mechanism of action of endogenous NPCs (Pluchino et al., 2005).

Whatever the therapeutic action exerted, transplanted NPCs show a certain degree of pathotropism toward inflammatory foci. This is due to the fact that such cells constitutively express an armamentarium of chemokines and chemokine receptors (e.g., CCR1, CCR5, CXCR3 and CXCR4), cell adhesion molecules (e.g., CD44) (Rampon et al., 2008) and integrins (e.g., VLA4) (Campos et al., 2004, 2006; Leone et al., 2008). Transplanted NPCs, very similarly to endogenous NPCs, have the characteristic to be able to follow and reach chemoattractant foci both when intraparenchymally and/or systemically injected (Ji et al., 2004; Pluchino et al., 2005).

When transplanted in acute or chronic inflammatory diseases (e.g., stroke, SCI, or MS), the majority of NPCs survive close to perivascular inflammatory foci (i.e., the atypical ectopic niche) where they interact with many other cell types such as CNS-infiltrating blood-borne inflammatory cells, endothelial cells and CNS-resident astrocytes and microglia. Within these ectopic niches, inflammatory molecules [e.g., interferon (IFN)γ, tumor necrosis factor (TNFα)] inhibit NPCs differentiation by blocking their cell cycle by up regulating the expression of cell cycle dependent kinase inhibitors (Pluchino et al., 2008). As undifferentiated cells, NPCs can produce a wide array of both secreted and transmembrane molecules which, in turn, exert both immunomodulatory and neurotrophic factors (Irvin et al., 2004; Pluchino et al., 2005; Seifert et al., 2005; Martino and Pluchino, 2006; Bacigaluppi et al., 2009; Cusimano et al., 2012).

In relapsing-remitting experimental autoimmune encephalomyelitis (EAE), the experimental model of MS, intravenously (i.v.) transplanted NPCs promote the apoptosis of encephalitogenic T cells either via the expression of death receptor ligands (for example, FasL, Trail and Apo3L) or the production of soluble mediators—i.e., NO synthase (iNOS), IFNγ—involved in mitochondrial-mediated apoptosis (Einstein et al., 2003, 2006; Pluchino et al., 2005). In the post-acute phase of ischemic or haemorrhagic stroke, i.v. transplantation of NPCs reduced activation of macrophage/microglia cells and CNS recruitment of blood-borne inflammatory cells (Lee et al., 2008; Bacigaluppi et al., 2009). Similarly, in the immediate time points following SCI, intrathecally (i.c.) as well as intralesionally transplanted NPCs modulate the local T cell, the microglial response (Ziv et al., 2006) and the recruitment of CNS infiltrating classically activated pro-inflammatory macrophages (Cusimano et al., 2012). Interestingly, it has been recently shown that also embryonic like induced pluripotent stem cell (iPSC)-derived NPCs—once transplanted intrathecally into mice with EAE—protect oligodendrocytes and OPCs from cell death. This transplantation promotes myelin tissue reconstruction via the selective production of leukemia inhibiting factor (LIF), and this production is guided by the inflammatory microenvironment (Laterza et al., 2013).

NPCs are therefore able to prevent inflammation-induced neuronal programmed cell death and glial scar formation—occurring, for example, in EAE, SCI, stroke—mainly via the paracrine secretion of the nerve growth factor (NGF), brain-derived neurotrophic factor (BDNF), ciliary neurotrophic factor (CNTF), and glial-derived neurotrophic factor (GDNF) (Teng et al., 2002; Lu et al., 2003; Pluchino et al., 2003, 2005; Chu et al., 2004; Ryu et al., 2004; Ziv et al., 2006; Redmond et al., 2007; Bacigaluppi et al., 2009).

Another bystander effect exerted by transplanted NPCs is to directly modulate neuronal circuit plasticity (Zhang and Chopp, 2009). In an experimental model of ischemic stroke human foetal NPCs significantly improved functional outcomes by promoting neuronal dendritic arborization in both hemispheres and axonal projections within the corticostriatal and corticospinal pathways. These effects have been attributed to the capacity of transplanted NPCs to re-express developmental molecules such as guidance molecules (i.e., slit, thrombospondin 1 and 2) but also trophic factor such as VEGF (Andres et al., 2011).

## Conclusions

NPCs in the adult brain exert an important homeostatic role either by producing new cells (neuronogenic or gliogenic function) or by orchestrating important processes (non-neurogenic functions): both actions are pivotal for the maintenance of the proper functioning of the CNS. Those neurogenic and non-neurogenic functions are in part NPC autonomous but are also driven by the microenvironment that might foster, according to the tissue needs, one of these functions. Our understanding of the complex interplay between neuronal, macroglial, and microglial cells in physiological and pathological conditions is continuously evolving, and we have now to consider NPCs as integral part of this interplay. New techniques of molecular biology and genetics will allow us to further understand the neurogenic vs. non-neurogenic functions of endogenous NPCs, and this knowledge would certainly help the scientific community to design efficacious stem cell-based treatment for still incurable neurological disorders.

### Conflict of interest statement

The authors declare that the research was conducted in the absence of any commercial or financial relationships that could be construed as a potential conflict of interest.
